# Mr. Guthrie's Lectures at the Royal College of Surgeons

**Published:** 1829-07-01

**Authors:** 


### XXXVI.

### ROYAL COLLEGE OF SURGEONS.

The annual lectures to be delivered in the theatre of the College by Professors Guthrie and Mayo, began on the 10th of March, when Mr. Guthrie delivered his introductory lecture. The first part he read, in order that no misapprehension might arise, as to whether the opinions expressed were his own, or those of any of his colleagues in the council. The latter was spoken, and without notes. The object of the written part was to urge the importance of anatomy and physiology, and inculcate the necessity for its cultivation on a broader basis, on a more extensive scale than that which is usually adopted. The following sentences seem to explain his views.

"In detaining you a short time on the subject of anatomy, it is not that I conceive you are unmindful of its importance, that you have not formed a true estimate of its value, that you are not prepared to assist in its cultivation to the extent of your ability; I am satisfied that each and every one I have the honor to address, is filled with admiration at the beautiful mechanism, the truths it discloses, the grand reflexions to which it leads, the submissive humility it inculcates, in demonstrating, as it were, with mathematical precision our nothingness, and the divine nature of the Supreme Being to whom we owe our formation."

"The development of truths of this nature, the aggregation of them so as to lead to any thing like system, is scarcely to be accomplished by the efforts of individuals, unless assisted by the influence, by the power of many. Some few individuals have arisen, who have made anatomy in its most comprehensive meaning, not only their peculiar study, but their pleasure, their amusement, their principal gratification. These men are an honor to the period in which they lived. They have proved inestimable benefactors of mankind, and have obtained for themselves that immortality of fame, which Nature, in her benevolence, had denied them of life. We have one great instance of this in the late Mr. Hunter, whose museum now adorns the walls of this College, forming at once their ornament and the monument of its collector:" and afterwards—"It is anatomy in its comprehensive sense, including every living thing on the earth, in the air, or under the waters, nay, closed up in the bowels of the earth, that is wanted, that I am advocating. It remains for some gifted, some powerful body (I would fain hope for this College) to institute such inquiries. Whenever it is done, on the great scale commensurate with the magnitude of the undertaking, it will be an act worthy of a scientific institution, and if a part of the funds, which have been silently accumulating for years, shall be devoted to such a purpose, then shall we all honor the prudence and foresight of those under whose management and care they had been collected. We shall respect the living, who have been unjustly defamed, whilst we regard with reverence those who are no more, but whose last wishes on earth were for the improvement of the art and science of surgery, and for the honor and prosperity of this College."

When Mr. Guthrie closed his book and addressed the President, he said his object was to excite, as Professor of Surgery, an earnest attention to its advancement; to shew, from the early history of the science, the means by which it had attained its present rank; what was wanting to add to it. After having gone through its rise and progress, he dwelt at length upon its present state, and called upon those who had raised themselves to the highest honors of the profession to go a step farther, to imitate Pitard, Vavassom and others of the olden time, who made their own advancement, their own interest, yield to that of their profession; who preferred its glory to their own. In the present day, he said, there was no profession, there was even no trade that was not represented in the House of Commons, save physic and surgery:—He earnestly inquired what there was so withering in the study of these sciences, which rendered a man less capable of bearing his part in public affairs, than if he had applied to the law, or to dealing in money, corn, bricks, leather, or any manufacture in which so many very eminent men were engaged. Surely, he said, physic and surgery could each find one man, whose locks were bleached by the hoar of sixty winters, rich in the worldly sense of the word, richer in the honors of the profession and in the good will of their contemporaries, who might, at a period like this, when it was attempted even to make laws for the profession, without its sanction, without its participation, stand forth as its champions.

We believe Mr. Guthrie here alluded to Sir H. Halford and Sir A. Cooper, and we cannot but agree with him that they ought to be in Parliament.

We cannot close this short account of these lectures, founded by Arris and Gall, without expressing our satisfaction at the manner in which they have been delivered. Mr. Guthrie possesses many excellent qualifications for a lecturer—a great command of language, with a clear and perspicuous mode of stating his subject; and however we might have expected that his practical experience would have afforded him abundant matter for practical lectures, we were not prepared to find that his professional attainments were so broad and deep; we must, therefore, congratulate the College in having selected one of their own council, who bids fair to uphold the high character which, from its first institution, it has derived from its anatomical and surgical professors.

